# Electrical stimulation promotes the angiogenic potential of adipose-derived stem cells

**DOI:** 10.1038/s41598-019-48369-w

**Published:** 2019-08-19

**Authors:** Jip Beugels, Daniel G. M. Molin, Daan R. M. G. Ophelders, Teun Rutten, Lilian Kessels, Nico Kloosterboer, Andrzej A. Piatkowski de Grzymala, Boris W. W. Kramer, René R. W. J. van der Hulst, Tim G. A. M. Wolfs

**Affiliations:** 1 Department of Plastic, Reconstructive and Hand Surgery, Maastricht University Medical Center+, Maastricht, 6229 HX The Netherlands; 2 NUTRIM School of Nutrition and Translational Research in Metabolism, Maastricht University, Maastricht, 6229 ER The Netherlands; 3 Department of Physiology, CARIM Cardiovascular Research Institute Maastricht, Maastricht University, Maastricht, 6229 ER The Netherlands; 4 Department of Pediatrics, Maastricht University Medical Center+, Maastricht, 6229 HX The Netherlands; 5 GROW School of Oncology and Developmental Biology, Maastricht University, Maastricht, 6229 ER The Netherlands; 6 MHeNs School for Mental Health and Neuroscience, Maastricht University, Maastricht, 6229 ER The Netherlands

**Keywords:** Angiogenesis, Mesenchymal stem cells, Stem-cell research

## Abstract

Autologous fat transfer (AFT) is limited by post-operative volume loss due to ischemia-induced cell death in the fat graft. Previous studies have demonstrated that electrical stimulation (ES) promotes angiogenesis in a variety of tissues and cell types. In this study we investigated the effects of ES on the angiogenic potential of adipose-derived stem cells (ASC), important progenitor cells in fat grafts with proven angiogenic potential. Cultured human ASC were electrically stimulated for 72 hours after which the medium of stimulated (ES) and non-stimulated (control) ASC was analysed for angiogenesis-related proteins by protein array and ELISA. The functional effect of ES on angiogenesis was then assessed *in vitro* and *in vivo*. Nine angiogenesis-related proteins were detected in the medium of electrically (non-)stimulated ASC and were quantified by ELISA. The pro-angiogenic proteins VEGF and MCP-1 were significantly increased following ES compared to controls, while the anti-angiogenic factor Serpin E1/PAI-1 was significantly decreased. Despite increased levels of anti-angiogenic TSP-1 and TIMP-1, medium of ES-treated ASC significantly increased vessel density, total vessel network length and branching points in chorio-allantoic membrane assays. In conclusion, our proof-of-concept study showed that ES increased the angiogenic potential of ASC both *in vitro* and *in vivo*.

## Introduction

Autologous fat transfer (AFT; also called fat grafting or lipofilling) is a widely used reconstructive and aesthetic procedure, wherein fat is harvested as an injectable filler to augment or reconstruct tissue in all regions of the body^1^. Currently, post-operative volume loss, described to be ranging from 40 to 80% of the initial graft volume^2–4^, is a significant limitation of AFT, and often requires additional grafting procedures. A lack of blood flow through the graft necessitates the transplanted cells to fully rely on the diffusion of oxygen and nutrients from surrounding tissue for their survival^5^. In an experimental mouse model, Kato *et al*.^6^ have shown that only adipocytes in the outermost layer of the transferred fat tissue survive the first week post-operative, while adipocytes present inwards of the diffusion limit of approximately 300 µm quickly die.

Numerous studies have focused on revascularization of the graft by stimulating angiogenesis in order to improve graft survival and maximize the retained graft volume. Approaches primarily focused on increasing the vascularization of the recipient site pre- and post-operatively^7,8^ or on modifying the pro-angiogenic potential of the adipose graft itself by adding single growth factors (i.e. FGF-2, PDGF and IL-8)^9–11^, platelet-rich plasma^12,13^, by adding regenerative cells from stromal vascular fractions^3,4^ or by adding isolated adipose-derived stem cells^14,15^. While administration of angiogenic growth factors has been proven to stimulate angiogenesis^16^, clinical implementation is hampered by the short serum half-life and adverse effects associated with continuous administration^17^. Others have suggested to enrich fat grafts with a heterogeneous population of regenerative cells, called the stromal vascular fraction, which is normally present in adipose tissue^18,19^. This procedure has been dubbed cell-assisted lipotransfer (CAL)^19^. However, Peltoniemi *et al*.^20^, did not find CAL to be superior to non-enriched fat grafts in respect to graft volume retention and for this reason concluded that regular AFT is cheaper, faster and has a lower risk of contamination. Adipose-derived stem cells (ASC) being abundantly present in adipose tissue are part of this stromal vascular fraction and have been studied for their angiogenic properties under numerous conditions^14,15,21–25^. In the only human clinical trial so far applying pure ASC, the authors isolated ASC from aspirated fat and expanded them *in vitro* for enriching the fat grafts that were injected in a second procedure^26^. Although ASC-enriched fat grafts remarkably retained 80.9% of the original graft volume (versus 16.3% for the controls), incorporating *ex vivo* expansion in daily clinical practice remains difficult because of its time and labour intensiveness, associated high costs and regulatory issues. Taking above mentioned limitations into account, a potential treatment which increases graft volume retention by stimulating revascularization in a cost-effective, non-invasive way is therefore essential to improve clinical care.

Previous *in vitro and in vivo* studies have demonstrated that electrical stimulation (ES) is able to stimulate angiogenesis in a variety of tissues and cell types^27–32^. Sheikh *et al*. demonstrated for example that ES of ischemic rabbit hind limbs significantly induced both arteriogenesis and angiogenesis^27^. The exact mechanisms remain to be elucidated, although in endothelial cells the enhanced release of VEGF and activation of VEGF receptors (VEGFRs), phosphatidylinositol-3-kinase (PI3K)-Akt and Rho-ROCK elements of the VEGFR signalling pathway play a significant role^29^. A major advantage of ES is that by influencing the secretion profile of stimulated cells it can induce autologous cells to secrete a myriad of important factors, in contrast of having to inject specific exogenous factors^30^. In light of these findings, we hypothesized that ES would enhance the secretion of paracrine angiogenic factors by ASC, leading to increased angiogenesis. In this study we aim to determine whether ES of ASC can stimulate *in vivo* parameters of angiogenesis.

## Results

### Determination of the electrical stimulus parameters

**Figure 1:**
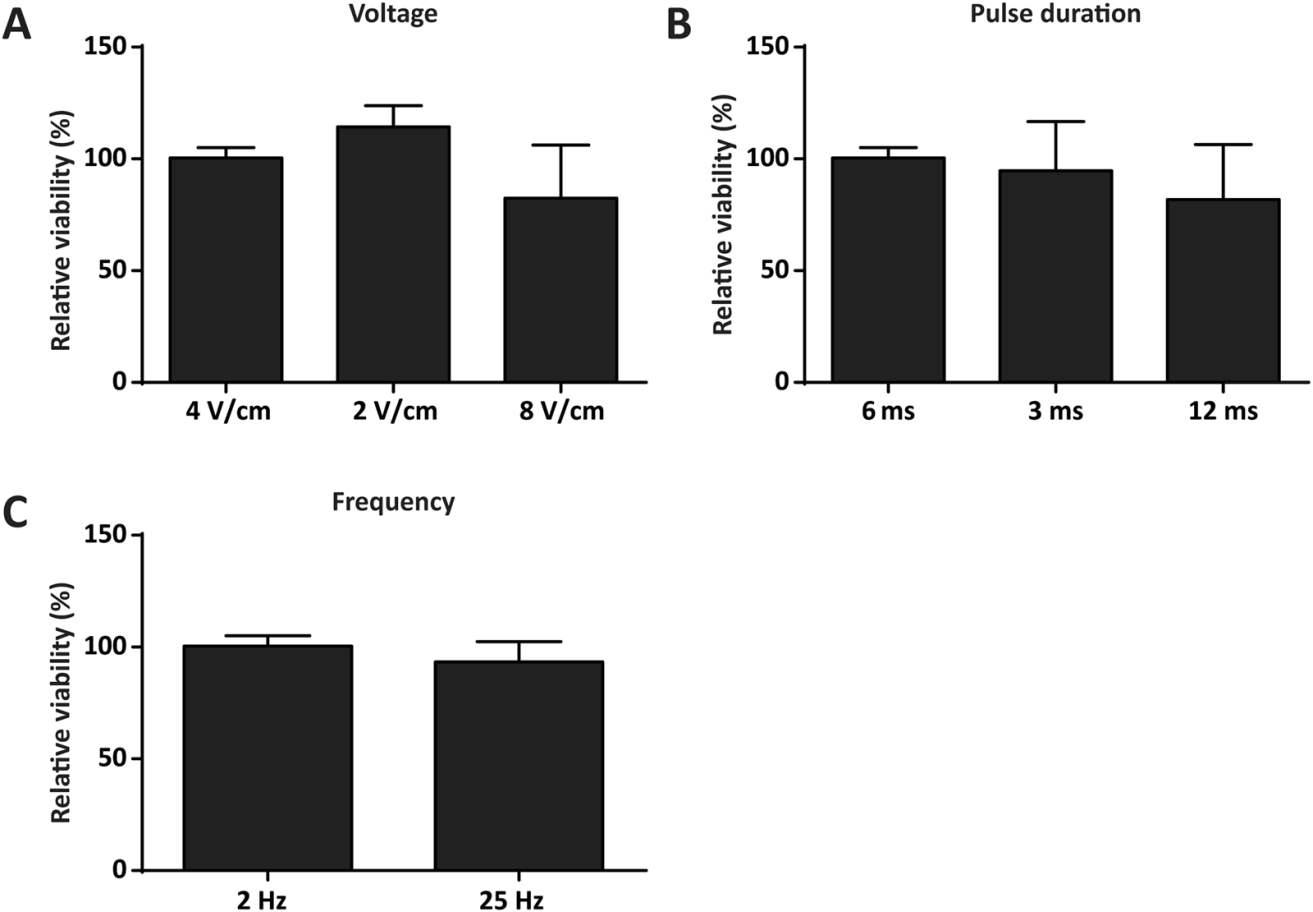
Determination of relative cell viability after electrical stimulation. Relative cell viability of electrically stimulated ASC for 72 hours versus unstimulated controls (CTRL set at 100%). Starting with a protocol of 4 V/cm, 6 ms pulses and 2 Hz, one parameter was changed per experiment while the other two parameters were kept at the original level. (**A**) Voltage was increased and decreased twofold. 4 V/cm had no effect on cell viability, whereas 2 V/cm increased viability and 8 V/cm decreased viability. (**B**) Pulse duration was set at 6 ms, 3 ms and 12 ms. Where 6 and 3 ms had no effect on cell viability, 12 ms decreased cell viability. (**C**) Only frequencies of more than tenfold the starting value showed a decrease in viability. Values are expressed as the mean percentage of relative viability (of ES vs. CTRL) + SD. n = 3 per parameter.

A titration experiment was performed to analyse the effect of different electrical stimulation parameters (i.e. voltage, pulse duration and frequency) on the viability and detachment of the ASC after 72 hours of continuous stimulation. Starting with 4 V/cm, 6 ms pulses at a frequency of 2 Hz, one parameter per experiment was increased or decreased (n = 3). High viability of the cells was detected for a maximum voltage of 4 V/cm and maximum pulse duration of 6 ms. Thereafter viability decreased versus unstimulated control (Fig. 1). The decrease in viability was paralleled by cell detachment in culture. Frequency had a lower impact on viability and a decreased viability was only seen when the frequency was set to the device’s maximum (25 Hz).

**Figure 2:**
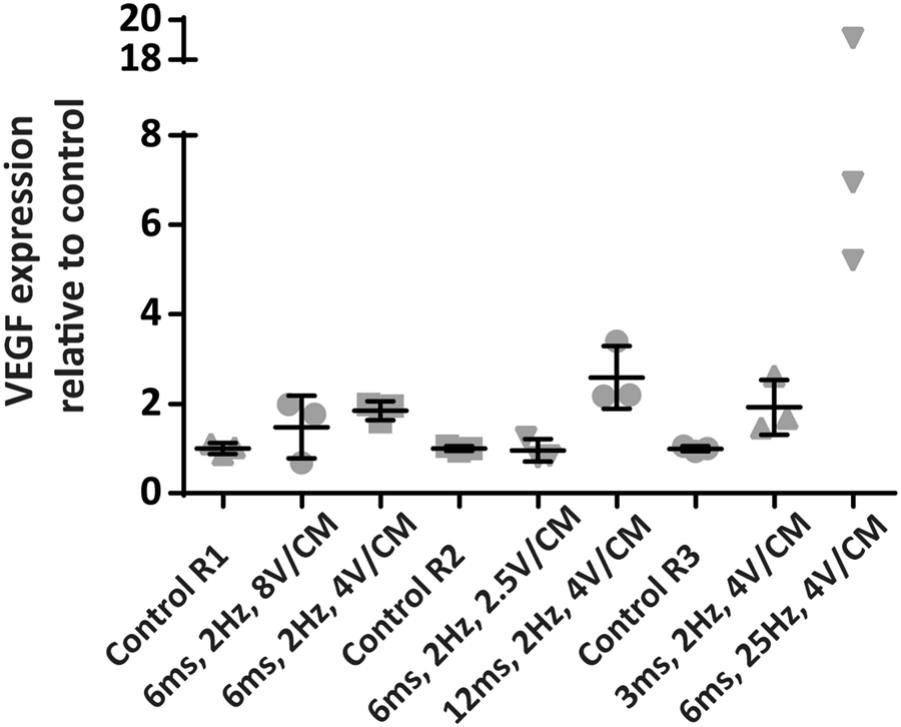
Three stimulation runs, each containing two ES groups and a control, were performed to analyse the effect of different treatment settings. Using 4 V/cm, 6 ms pulse duration and 2 Hz a 1.85 times increase in VEGF concentration was found compared to non-stimulated control. Elevated levels of VEGF were also found when we increased/decreased voltage. In the elevated pulse duration and frequency groups, cells were substantially detached which was in part reflected by increased cell death, especially in the 25 Hz group, explaining the highly increased VEGF A expression. Values are expressed as the mean VEGF expression relative to control. N = 3 per stimulation protocol.

An additional experiment was conducted to investigate the effect of different electrical stimulation parameters on the concentration of VEGF A in the conditioned medium as a key component of the angiogenic process (Fig. 2). Using 4 V/cm, 6 ms pulse duration and 2 Hz we found a 1.85 times increase in VEGF concentration compared to the non-stimulated control. Elevated levels of VEGF were also found when we increased voltage (factor 1.48 at 8 V/cm), pulse duration (factor 2.59 at 12 ms) and frequency (factor 10.41 at 25 Hz). However, in case of the elevated voltage and pulse duration groups, cells were substantially detached which was even in part reflected by increased cell death. This phenomenon was especially prevalent when ASC were exposed to the highest frequency, explaining the relatively high VEGF concentration in this group. When the pulse duration was decreased to 3 ms, a 1.92 times higher VEGF concentration was found when compared to unstimulated cells. However, the inter group variation between concentrations was higher when compared to the cells exposed to 4 V/cm, 6 ms, 2 Hz. Importantly this latter protocol was also used as preferred setting in a previous publication^33^. Based on the group homogeneity and historical data, the parameters 4 V/cm, 6 ms and 2 Hz were used as standard setting for the study.

### Conditioned medium of electrically stimulated adipose-derived stem cells contains multiple pro- and anti-angiogenic factors

**Figure 3:**
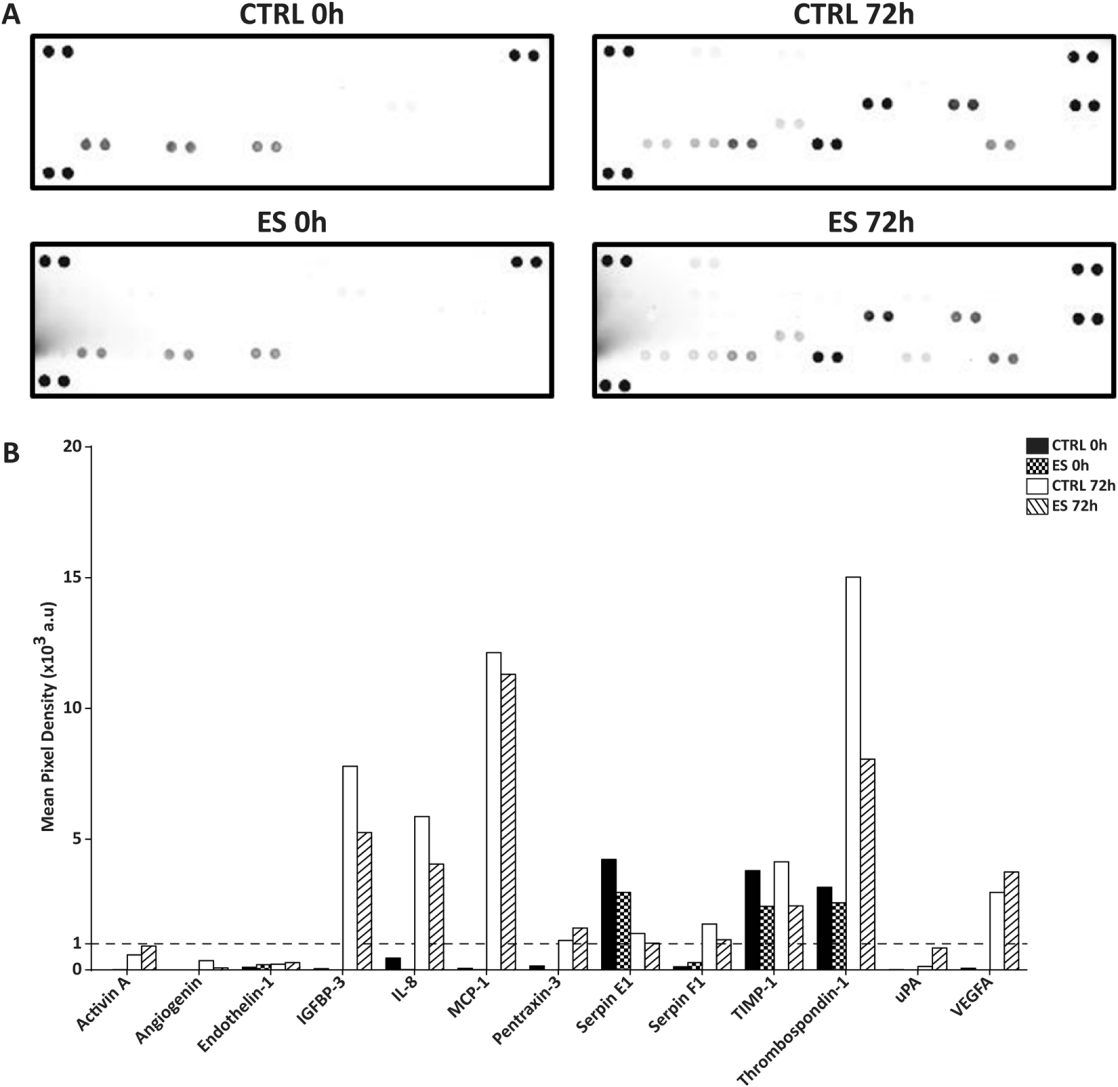
Screening of conditioned medium using a human angiogenesis proteome profiler array. (**A**) The respective membranes are depicted for CTRL and ES 0 h and 72 h, respectively. Next to the positive array controls in upper left, upper right and lower left corners, the in duplo spots for a total of 13 proteins were visible after scanning. (**B**) Mean pixel density was calculated for each spot, after normalization to the background. Nine proteins reached a minimum mean pixel density of 1 × 10^3^ arbitrary units (a.u.; dotted line). For each group a single medium sample was analysed.

Medium of both ES and CTRL ASC cultures for time points 0 h (baseline) and 72 h were screened for the presence of pro- and anti-angiogenic factors using a human angiogenesis proteome profiler array (Fig. 3A), which has been used for ASC before^34^. The presence of 13 separate proteins was detected in the conditioned medium of ES and CTRL 72 h, of which 9 proteins (IGFBP-3, IL-8, MCP-1, PTX-3, SERPIN-E1, SERPIN-F1, TIMP-1, TSP-1 and VEGF-A) were above the pixel density threshold of 1 × 10^3^ arbitrary units (Fig. 3B). No difference between control and ES group in terms of baseline medium was found. Regarding the typical semi-quantitative nature of the protein profiler array, no conclusions were drawn for differential signal between the groups. Rather, the proteins above threshold were assigned for further quantitative analysis.

### Electrical stimulation increases both pro- and anti-angiogenic proteins in ASC medium

**Figure 4:**
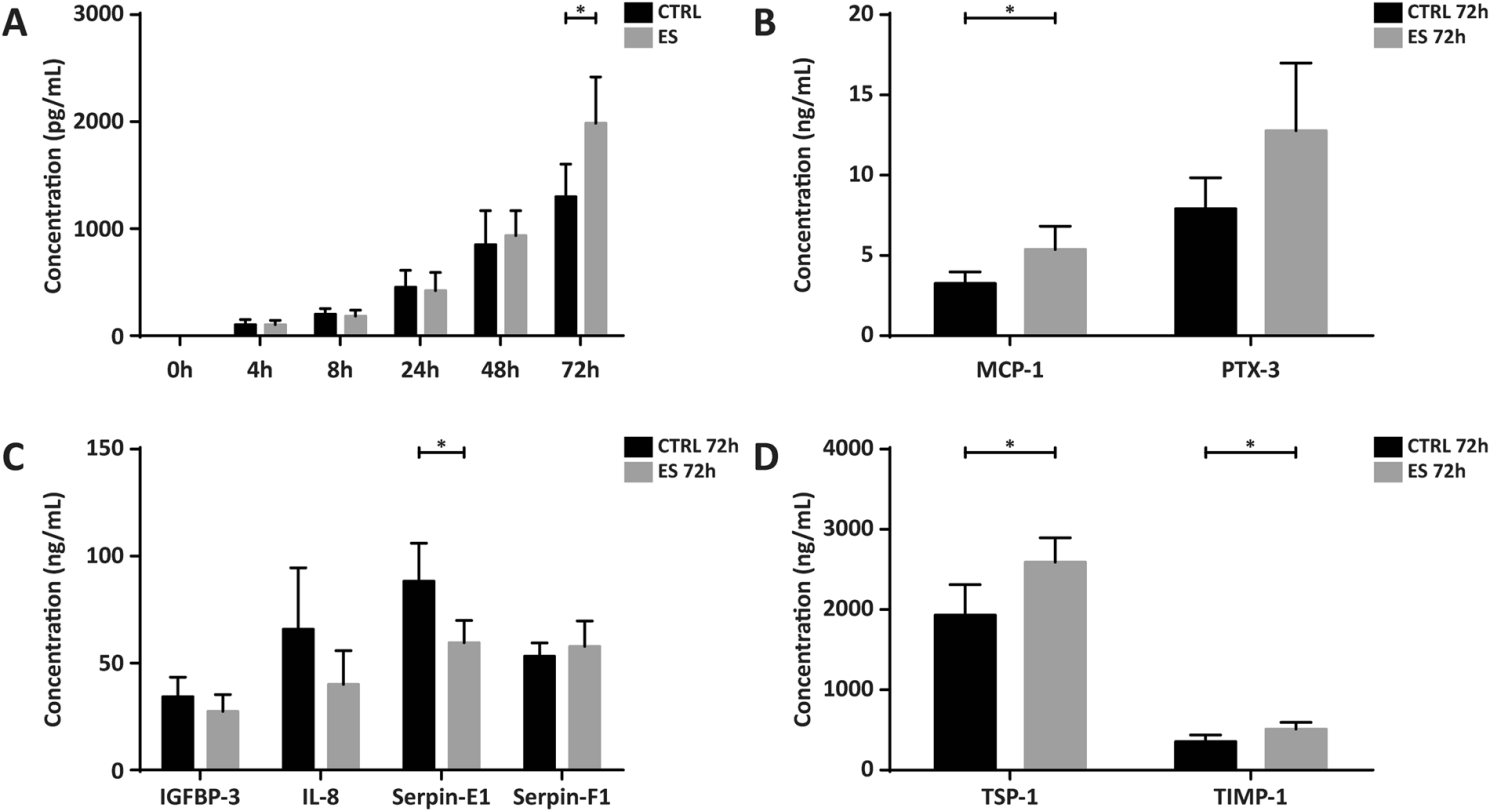
Quantification of the identified proteins with ELISA. (**A**) Concentrations of VEGF in the CM over time, ES vs. CTRL. The concentration at 72 h was significantly different for ES compared to CTRL (p = 0.04). The gradual rise in concentration visible in the time curve was representative for that of the other proteins. (**B**) MCP-1 was significantly increased in ES vs CTRL (p = 0.04), whereas the increase in PTX-3 did not reach statistical significance (p = 0.08). (**C**) Serpin-E1 was significantly decreased in ES vs CTRL (p = 0.03), whereas IGFBP-3, IL-8 and Serpin-F1 were not statistically different. (**D**) TSP-1 and TIMP-1 were both significantly increased in ES vs. CTRL (p = 0.03 and p = 0.04, respectively). Values displayed are mean + SD. *p < 0.05. n = 4 per group.

To confirm and quantify differential expression of the identified proteins, ELISA were performed on baseline 0 h medium and on the CM of 4h-72h for each of the 9 proteins. Protein levels in the CM showed an increase parallel to post-stimulation time, with no apparent differences between CTRL and ES for the first 48 h (Fig. 4A; depicted for VEGF-A). All secreted proteins analysed revealed a similar pattern with a gradual increase in concentration over time. At 72 h significant differences between ES and CTRL were detected for several proteins (Fig. 4B); VEGF A (1.99 vs. 1.30 ng/mL; p = 0.04), TSP-1 (1,93 v. 2,59 ng/mL; p = 0.03), TIMP-1 (356.50 vs. 512.90 ng/mL; p = 0.04), and MCP-1 (3.26 vs. 5.38 ng/mL; p = 0.04) were significantly increased in the ES group vs CTRL, whereas Serpin-E1 was significantly decreased in the ES group vs CTRL (59.6 vs 88.3 ng/mL; p = 0.03). PTX-3 was increased in the ES group vs. control (7.90 vs. 12.77 ng/mL), however the difference did not reach statistical significance (p = 0.08). Since only the 72 h medium showed significant differences in protein secretion between groups, medium of this time point was used in subsequent functional assays.

### Conditioned medium of electrically stimulated ASC induced angiogenesis *in vivo*

**Figure 5:**
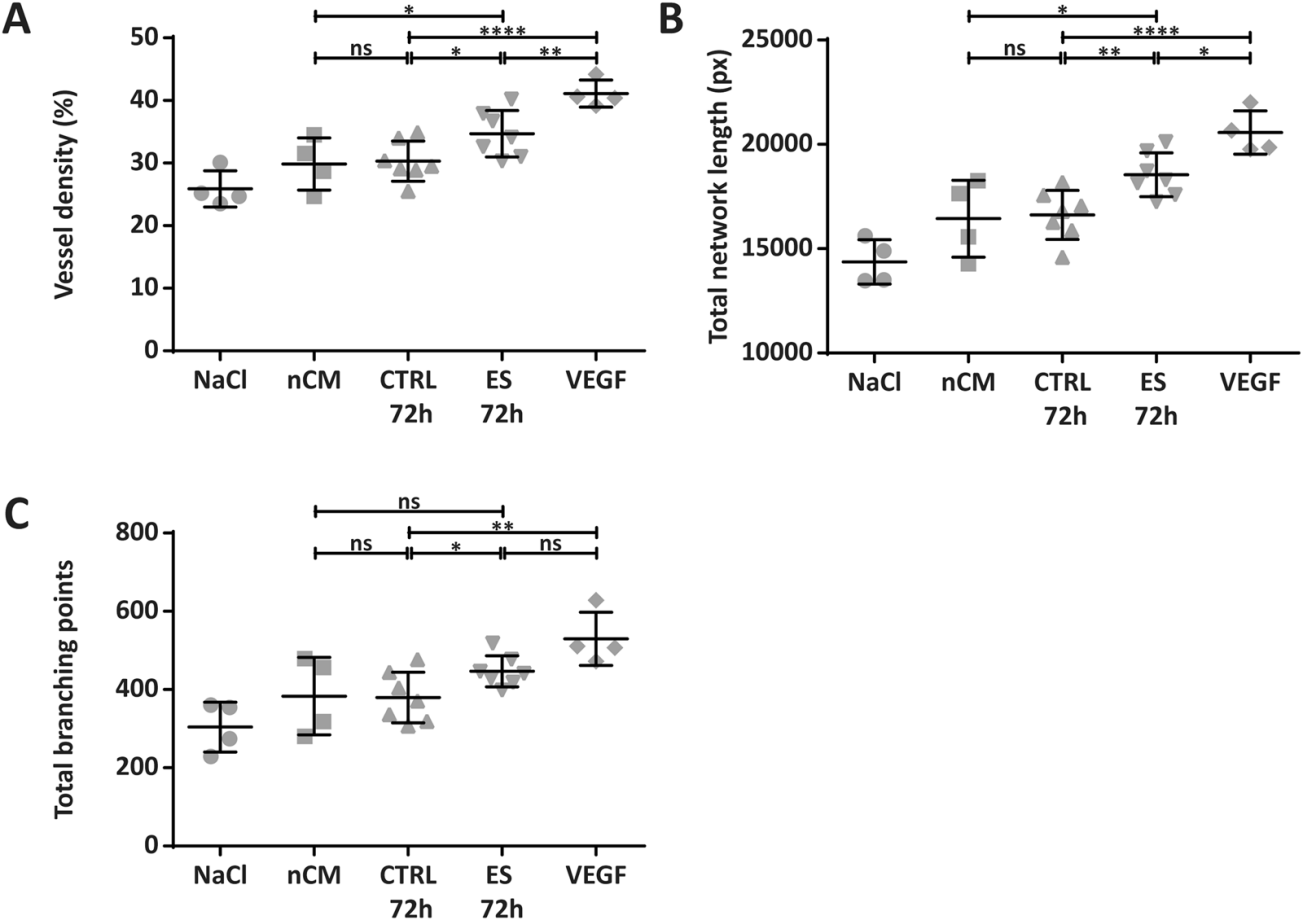
Conditioned medium of electrically stimulated ASC induced angiogenesis *in vivo*. ES CM significantly increased vessel density **(A)**, total vessel network length **(B)** and total number of branching points **(C)** compared to CTRL CM in a chorioallantoic membrane (CAM) assay. *p < 0.05; **p < 0.01; ****p < 0.0001. n = 4–7 CAM per group. px = pixel, ns = not significant, NaCl = 0.09% sodium chloride as negative control, nCM = non-conditioned baseline medium, CTRL 72 h = non-stimulated conditioned medium after 72 h of incubation, ES 72 h = electrically stimulated conditioned medium after 72 hours, VEGF = positive control.

To determine whether the observed changes in angiogenesis-related proteins after electrical stimulation induced an angiogenic effect *in vivo*, a CAM assay was performed. In the ES group compared to controls, a significant increase in density of the vessels within the ring was detected (34.7 vs 30.3%; p < 0.05), as well as an increased total vessel network length (18,541 vs. 16,621 px; p < 0.01) and a higher total number of branching points (447 vs. 380; p < 0.05) (Fig. 5). Representative images of the control and ES-treated CAM, including their analysis, can be found in Supplementary Fig. S1.

## Discussion

This proof-of-concept study demonstrates that ES of ASC improves *in vitro* and *in vivo* parameters of angiogenesis. Using a protocol of continuous stimulation with a biphasic electrical current of 4 V/cm, 6 ms pulse duration with a frequency of 2 Hz, we detected significant differences in ASC secreted protein level for several pro- and anti-angiogenic proteins after 72 hours of ES. ES of ASC significantly augmented the concentrations of the pro-angiogenic proteins VEGF-A^35^ and MCP-1^36^, whereas the anti-angiogenic protein Serpin E1/PAI-1^37^ was significantly decreased compared to non-stimulated controls. The elevated level of VEGF confirms and extends previous work from Tandon *et al*.^38^, showing increased *VEGF-A* mRNA expression after ES of ASC. The concentrations of VEGF found in the conditioned medium range from an average of 1.30 ng/mL for the control group to an average of 1.99 ng/mL for the ES group, representing a 153% increase in VEGF. This increase seems biologically relevant since the concentrations of 1.30 and 1.99 ng/ml fit exactly in the biologically relevant range of concentrations, reported to stimulate endothelial cells *in vitro*^39^. A robust increase of *VEGF-A* mRNA expression and/or VEGF-A protein level in response to ES is also supported by studies focusing on various other human cell types, such as endothelial cells^29^, bone marrow-derived mesenchymal stromal cells^30^, cardiomyocytes^40^, osteoblasts^41^ and skeletal muscle cells^42^. Although an influence of ES on the secretion of MCP-1 was shown for macrophages^43^, no reports have been made on a similar effect for ASC. Only for ASC stimulated with the pro-inflammatory stimulus TNF-α an increase of MCP-1 secretion has been shown^44,45^. Comparably, TNF-α-induced upregulation has been reported for Serpin E1/PAI-1^44,45^, whereas we provide data showing the opposite to occur after ES. Taken together, these findings suggest that ES is not the equivalent of a pro-inflammatory stimulus, a finding which is important for clinical translation where the amplification of the pro-inflammatory environment post-operative must be avoided. In addition to the pro-angiogenic factors described above, ES significantly induced the release of two anti-angiogenic factors, namely TSP-1^46^ and TIMP-1^47^. Under physiological circumstances pro- and anti-angiogenic stimuli are well balanced and related feedback mechanisms are considered to prevent unrestricted growth of blood vessels^48^. In this study, we found a positive pro-angiogenic effect *in vivo* using the conditioned medium, even though two inhibitors of angiogenesis were upregulated in the ES medium. Inhibition of TSP-1 and TIMP-1 secretion in future experiments might induce an even more profound pro-angiogenic response.

Using the CAM assay we found a pro-angiogenic effect, as net result of the altered pro- and anti-angiogenic factors. The CAM assay allows analysis of the full blown angiogenic potential of stimuli, ranging from tubulogenesis up to stabilization of the vessel over a longer period of time. The higher cellular complexity of the CAM assay (i.e. endothelial cells and pericytes) together with the options to test the angiogenic capacity of stimuli for the different phases of angiogenesis provides in depth information on the angiogenic potential. Reviewing the results of Kim *et al*.^30^ who reported a small but statistically significant increase of VEGF after electrical stimulation of BM-MSC after 1 day and a more profound effect after 2 days, we can conclude that several factors (e.g. cell type or ES protocol) influence the timeframe in which statistically significant differences between control and ES can be detected. This has important clinical implications since the majority of transplanted adipocytes in the hypoxic region of the graft die within the first 24 hours after transplantation^5^. Starting with ES of the donor and recipient site several days pre-operative in order to create a pro-angiogenic environment at the time of surgery is one way to utilize the effect of ES in the short window-of-opportunity. Alternatively, future studies should evaluate the effect of altering key stimulus parameters such as wave form, voltage, pulse duration and frequency to assess which ES protocol provides the earliest pro-angiogenic response. More precisely, Zhao *et al*.^49^ found significantly elevated levels of VEGF as early as 5 minutes after ES of endothelial cells using their custom built ES set-up. Rackauskas *et al*.^40^ evaluated different ES regimes for cardiomyocytes and found a frequency-dependent effect, both on the level of secreted VEGF protein in the medium and on the growth of cultured human coronary artery endothelial cells treated with CM from stimulated cells.

While various forms of ES, such as direct or alternating current (DC or AC)^38,50–52^, capacitive coupling (CC)^53^, and pulsed electromagnetic fields (PEMF)^54,55^ have been used to stimulate ASC, each have their advantages and disadvantages. In case of DC stimulation, treatments can be separated in two main groups: constant or pulsed electric stimulation. Whereas the former leads to a build-up of charge causing electrolysis and hence changes in pH and oxygen tension around the cathode, accumulation of proteins and necrosis *in vivo*^30,56^, we chose biphasic current to circumvent these side effects due to charge balance resulting from the bidirectional wave consisting of a positive and negative phase. In addition, biphasic waveforms are often referred to as most comfortable to the patient with the least skin reaction^57^.

With the establishment of proof-of-concept for increased angiogenesis after stimulation of ASC, the question arises what the influence of ES would be on other angiogenic cells contained within fat. We envision a non-invasive, cost-effective and easy clinical approach to electrically stimulate adipose tissue *in vivo* by applying biphasic electric current from a portable stimulator through electrodes attached to the skin. Multiple treatments such as electrical muscle stimulation (EMS) and transcutaneous electrical nerve stimulation (TENS) have already used this set-up for years. Using these devices it is possible to choose from a wide range of stimuli and create an optimal treatment schedule that can include both pre- and post-operative stimulation, without the need to isolate cells and treat them *in vitro*.

In conclusion, our proof-of-concept study showed that ES increased the angiogenic potential of ASC as demonstrated with *in vitro* and *in vivo* parameters. The results provide a valid basis for a translational study in which the effect of a non-invasive ES intervention will be analysed in a pre-clinical model of AFT.

## Materials and Methods

### Cells

Human ASC were purchased from Lonza (Basel, Switzerland. Single donor: 52Y female, BMI 24). After thawing, ASC were seeded at a density of 5,000 cells/cm^2^ in culture medium consisting of low glucose Glutamax Dulbecco’s modified Eagle medium (DMEM, Thermo Fisher Scientific, MA, USA), supplemented with 10% MSC-qualified fetal bovine serum (FBS, Thermo Fisher Scientific) and 1% penicillin/streptomycin. ASC were subcultured according to the manufacturer’s protocol until reaching 90% confluence, and used at passage 5 in all experiments.

### Electrical stimulation

Cells were stimulated using the C-pace EP Cell culture stimulator (Ion-Optics Co., MA, USA) to generate a biphasic electrical current. As described previously^33^ cells were electrically stimulated using a protocol of 4 V/cm with 6 ms pulses at a frequency of 2 Hz. However, considering this study focused on ES of a different cell type (i.e. ASC), a dose-response curve was performed to determine the effect of different stimulation protocols on cell viability and Vascular Endothelial Growth Factor (VEGF A) release. Starting with the above mentioned parameters of 4 V/cm, 6 ms pulses and 2 Hz in each subsequent stimulation round, one of the parameters (i.e. voltage) was increased or decreased, while the other two parameters were kept at the original setting. The investigated settings were as follows:

1. 2 V/cm, 6 ms and 2 HZ
2. 4 V/cm, 6 ms and 2 Hz
3. 8 V/cm, 6 ms and 2 Hz
4. 4 V/cm, 3 ms and 2 Hz
5. 4 V/cm, 12 ms and 2 Hz
6. 4 V/cm, 6 ms and 25 Hz

ASC were cultured in 6-well plates (TC treated, Greiner bio one, Kremsmünster, Austria) at 15,000 cells/cm^2^. Stimulated cell cultures were checked for cell adherence and overall morphology. After 72 hours of continuous stimulation, cell number and viability were assessed using a cell counter with integrated Trypan Blue detection (Bio-rad, CA, USA).

### Conditioned medium

Baseline medium (t0) and conditioned medium (CM) were collected each from a different well at the following time points: 4, 8, 24, 48 and 72 hours (t4-t72). To this end, ASC were seeded at 15,000 cells/cm^2^ in 6 well plates in culture medium and allowed to adhere to the plate for 12 hours. Next, cells were starved by culturing them in medium with 5% serum for 12 hours and before stimulation “starvation” medium was refreshed completely. After centrifugation for 5 minutes at 300 g the CM was aliquoted and stored at −20 °C before use in subsequent experiments.

### Angiogenesis array

To determine the production of angiogenesis-related proteins of stimulated and control samples, a human angiogenesis proteome profiler array which enables detection of 55 angiogenesis-related proteins per sample was used according to the manufacturer’s instructions (R&D Systems Inc., MN, USA). Near infrared fluorescence detection with IRDye 800CW (Li-cor, NE, USA) was applied and signals were visualized by using an Odyssey CLx imager (Li-cor). After normalization to the background, average pixel intensity of each of the spots’ pixels within a fixed circular boundary was determined using image analysis software (Odyssey v1.2, Li-cor).

### Enzyme-linked immunosorbent assays

For data confirmation of the array and quantification of the angiogenesis-related proteins with an average pixel intensity of ≥1.0^3^ arbitrary intensity units^58^ as defined with the angiogenesis array, enzyme-linked immunosorbent assays (ELISA) were performed. Levels of Vascular Endothelial Growth Factor A (VEGF A), urokinase-type Plasminogen Activator (uPA), Insuline-like Growth Factor Binding Protein (IGFBP-3), Interleukin 8 (IL-8), Monocyte Chemoattractant Protein 1 (MCP-1), Pentraxin-3 (PTX-3, also known as TSG-14), Plasminogen Activator Inhibitor 1 (PAI-1 also known as Serpin E1), Pigment Epithelium-derived Factor (PEDF, also known as Serpin F1), Thrombospondin-1 (TSP-1) and TIMP Metallopeptidase Inhibitor (TIMP-1) were measured in the CM of ES and Control groups for each respective time point, using Duoset ELISA kits (R&D Systems Inc.) according to the manufacturer’s instructions. The OD’s were measured at a wavelength of 450 nm using a Thermo Multiskan Spectrum plate reader and SkanIt software (Thermo Fisher Scientific).

### Chorioallantoic membrane assay

The protocol for the chorioallantoic membrane (CAM) assay was based on the procedure described by Le Noble *et al*.^59^. Fertilized White Leghorn eggs were incubated in an egg incubator for 3 days at 37.8 °C and a relative air humidity of 55%, while being rotated every hour. At day 3 a rectangular window measuring 1 × 1.5 cm was cut into the eggshell. Initially, the eggshell membrane was left intact to prevent any debris from the shell falling into the egg. After making a small hole in the eggshell membrane, two milliliters of albumin were withdrawn from the blunt end of the egg, using a 21 G needle at an angle of 45 degrees. Care was taken to prevent sticking of the embryo against the membrane. Next, the membrane was removed to expose the embryo. Subsequently, the window was covered with adhesive tape to prevent dehydration. The eggs were placed back in the incubator without rotation until day 7 when a small silicon ring, measuring 1 cm and weighing 17 mg was placed on the developing CAM. Sixty-five microliters of each respective test solution was injected inside the ring on the membrane of each egg after filtering of the solution with a 0.2 micrometer syringe filter. The test solutions consisted of sodium chloride 0.9% (negative control), non-conditioned medium (nCM, consisting of DMEM, 5% FCS and 1% PS), conditioned medium from the control group that was incubated for 72 hours (CM CTRL), conditioned medium after 72 hours of stimulation (CM ES) and VEGF (positive control, dissolved in DMEM, at a total daily dose of 20 ng/mL of VEGF). Test solutions were applied daily until day 14. At day 14 the shell was opened to expose the CAM. In order to increase the contrast and to isolate the blood vessels in the CAM from vessels running over the yolk, a suspension of zinc oxide in vegetable oil was injected right underneath the ring and its surroundings. A Leica MS5 stereomicroscope, Leica IC A camera and VideoVelocity software (Candylabs, Vancouver, Canada) were then used to photograph the area within the ring at a magnification of 4×. The images were then analysed using the objective and completely automated Wimasis image analysis service (WimCAM, OnImagin Technoglogies SCA, Cordoba, Spain)^60^. Only eggs with no major blood vessels inside the ring were included, to decrease intra- and inter-group variation. Total vessel density (in %), total vessel network length (in px) and total branching points were extracted from the analysed data.

### Statistical analyses

Data are expressed as the mean ± standard deviation (SD). ELISAs were performed in duplicate over four independent CM samples (n = 4). In the tube assay the n value ranged from 3–5. For the CAM assay at least four eggs per condition were included (n = 4–7). The ELISA results were analysed by unpaired Student t-test, whereas one-way ANOVA with Bonferroni’s multiple comparisons test was used to analyse the tube assay and CAM assay data (GraphPad Prism 6, GraphPad Software Inc., San Diego, USA). A p-value < 0.05 was considered statistically significant.

## Supplementary information

Supplementary figure S1

## Data Availability

All data generated or analysed during this study are included in this published article.
