# Supplementary material for: Electrical stimulation promotes the angiogenic potential of adipose-derived stem cells
Source: Sci Rep. 2019 Aug 19;9:12076. doi: 10.1038/s41598-019-48369-w (PMC6700204; doi:10.1038/s41598-019-48369-w)
Supplement: Supplementary file 1 — Supplementary figure S1 [file 41598_2019_48369_MOESM1_ESM.pdf]

Electrical stimulation promotes the angiogenic potential of adipose-derived stem cells

Jip Beugels[1,2], Daniel G.M. Molin[3], Daan R.M.G. Ophelders[4,5], Teun Rutten[4], Lilian Kessels[4], Nico Kloosterboer[4], Andrzej A. Piatkowski de Grzymala[1,2], Boris W.W. Kramer[4,5,6], René R.W.J. van der Hulst[1,2], Tim G.A.M.Wolfs[4,5*]

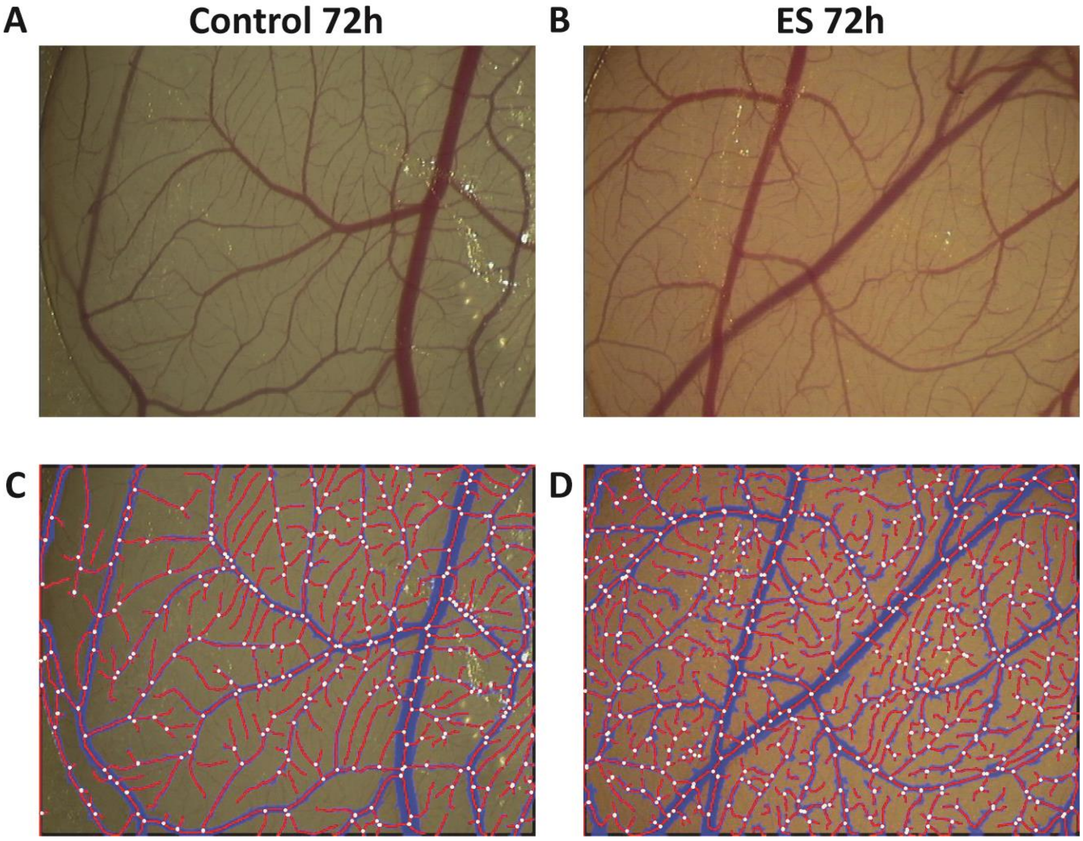

Supplementary figure S1. Representative images of the ES and CTRL CAM at day 14. (A): contrast agent was injected under the CAM to enhance the identification of capillaries before photographs were made. **(B):** lower panels reflect the computer analyzed overlays, wherein all vessels are tracked by automated Wimasis image analysis service.
